# Probiotics and atherosclerosis – a new challenge?

**DOI:** 10.3402/mehd.v23i0.18576

**Published:** 2012-06-18

**Authors:** Chan Yee Kwan, Pirkka Kirjavainen, Chen Yan, Hani El-Nezami

**Affiliations:** 1 School of Biological Sciences, University of Hong Kong, HKSAR, Hong Kong, China; 2 Institute of Public Health and Clinical Nutrition, University of Eastern Finland, Kuopio, Finland; 3 Department of Surgery, University of Hong Kong, HKSAR, Hong Kong, China; 4 Department of Chemistry, University of Hong Kong, HKSAR, Hong Kong, China

**Keywords:** ApoE^−/−^ mice, LGG, VSL#3, gut microbiota, cardiovascular disease

## Abstract

**Background:**

Atherosclerosis is the major cause of cardiovascular disease and stroke, which are among the top 10 leading causes of death worldwide. Pathogen-associated molecular patterns (PAMPs) can activate toll-like receptors (TLRs) and activate nuclear factor kappa B (NFκB) signaling, a central pathway in inflammation, which regulates genes that encode proinflammatory molecules essential in atherogenesis. Lipopolysaccharides (LPS), which is unique to gram negative bacteria, as well as peptidoglycan (PGN), which is found in gram positive bacteria are PAMPS and ligands of TLR4 and TLR2, respectively, both of which are essential in plaque progression in atherosclerosis. Gastrointestinal tract is suggested to be the major site for absorption and translocation of TLR2 and TLR4 stimulants. Inflammation can result in a ‘leaky gut’ that leads to higher bacterial translocation, eventually the accumulation of LPS and PGN would activate TLRs and trigger inflammation through NFκB and promote further systemic complication like atherosclerosis. Probiotics, can protect the intestinal barrier to reduce bacterial translocation and have potential systemic anti-inflammatory properties.

**Objective:**

To evaluate whether probiotics can help reduce atherosclerotic development using *in vivo* study.

**Design:**

Apolipoprotein E knockout (ApoE^−/ −^) mice were fed on high fat diet alone, with telmisartan (Tel) (1 or 5 mg/kg/day, positive controls) or with probiotics (VSL#3/LGG) with or without Tel (1 mg/kg/day) for 12 weeks.

**Results:**

Probiotics, Tel, or a combination of both reduced lesion size at the aortic root significantly; VSL#3 reduced serum inflammatory adhesion molecules soluble E- (sE-)selectin, soluble intercellular adhesion molecule 1 (sICAM-1), soluble vascular cell adhesion molecule 1 (sVCAM-1), and plaque disrupting factor matrix metalloproteinase (MMP)-9 significantly; probiotics and Tel at 5 mg/kg/day could induce changes in gut microbiota population; the efficiency of lesion reduction seemed to correlate to the microbiota composition; probiotics seemed to reduce plasma endotoxin but did not reach statistical significance.

**Conclusion:**

Probiotics has the potential to be used as a cheap, non-invasive, and with little side effects way to reduce atherosclerosis that brings worldwide benefits.

